# Naphthoquinone Tryptophan Hybrids: A Promising Small Molecule Scaffold for Mitigating Aggregation of Amyloidogenic Proteins and Peptides

**DOI:** 10.3389/fcell.2019.00242

**Published:** 2019-10-17

**Authors:** Guru KrishnaKumar Viswanathan, Ashim Paul, Ehud Gazit, Daniel Segal

**Affiliations:** ^1^Department of Molecular Microbiology and Biotechnology, School of Molecular Cell Biology and Biotechnology, Tel Aviv University, Tel Aviv, Israel; ^2^Interdisciplinary Sagol School of Neurosciences, Tel Aviv University, Tel Aviv, Israel

**Keywords:** amyloid aggregation, Naphthoquinone Tryptophan hybrids, peptides and proteins, self-assembly inhibitors, small molecule

## Abstract

A current challenge faced by researchers is the lack of disease-modifying therapeutics for amyloid formation that is associated with several human diseases. Although the monomeric proteins or peptides involved in various amyloidogenic diseases do not have amino acid sequence homology, there appears to be a structural correlation among the amyloid assemblies, which are responsible for distinct pathological conditions. Here, we review our work on Naphthoquinone Tryptophan (NQTrp) hybrids, a small molecule scaffold that can generically modulate neuronal and non-neuronal amyloid aggregation both *in vitro* and *in vivo*. NQTrp reduces the net amyloid load by inhibiting the process of amyloid formation and disassembling the pre-formed fibrils, both in a dose-dependent manner. As a plausible mechanism of action, NQTrp effectively forms hydrogen bonding and hydrophobic interactions, such as π-π stacking, with the vital residues responsible for the initial nucleation of protein/peptide aggregation. This review highlights the effectiveness of the NQTrp hybrid scaffold for developing novel small molecule modulators of amyloid aggregation.

## Introduction

Quinones are an important class of organic molecules, composed of cyclic diones, six membered rings with two carbonyls conjugated to double bonds of the cyclic structure, and play a pivotal role in cellular functions (Nohl et al., 1986). They are found as an integral part of numerous natural products and exhibit significant biological properties such as anticancer, antibiotic, antioxidant, trypanocidal, and antimalarial activities (Rohr and Thiericke, 1992; Pinto and de Castro, 2009; Sunassee and Davies-Coleman, 2012; Li et al., 2015). In the past two decades, various quinones and their derivatives have been identified as potential therapeutic molecules toward amyloid-associated diseases including Alzheimer’s disease (AD), Parkinson’s disease (PD) and Type-2 Diabetes mellitus (T2DM) (Li et al., 2004; Pickhardt et al., 2005; Convertino et al., 2009; Gong et al., 2014).

Several types of quinones including benzoquinones (BQ), naphthoquinones (NQ), anthraquinones (AQ), and phenanthraquinones (PQ) exhibit notable anti-amyloidogenic properties toward the causative proteins/peptides involved in various protein misfolding diseases via specific mechanisms (Scherzer-Attali et al., 2010; Gong et al., 2014; Brahmachari et al., 2017; Viswanathan et al., 2018). For example, the smallest quinone member, 1,4-benzoquinone was shown to inhibit aggregation of the hen egg-white lysozyme (Lieu et al., 2007) and 1,4-naphthoquinone was demonstrated as a lead molecule toward amyloids associated with various neurodegenerative diseases (Tomiyama et al., 1996; Bermejo-Bescos et al., 2010). Likewise, 9,10-anthraquinone was found to effectively inhibit fibrillization of the Tau protein and Aβ peptide, major culprits of AD (Pickhardt et al., 2005; Convertino et al., 2009). Pyrroloquinoline quinone inhibited amyloid formation by Aβ, α-synuclein and the prion protein (Kobayashi et al., 2006; Zhang et al., 2009; Kim et al., 2010), whereas, a quinone derivative of dopamine inhibited α-synuclein fibrillization (Bisaglia et al., 2010).

**FIGURE 1:**
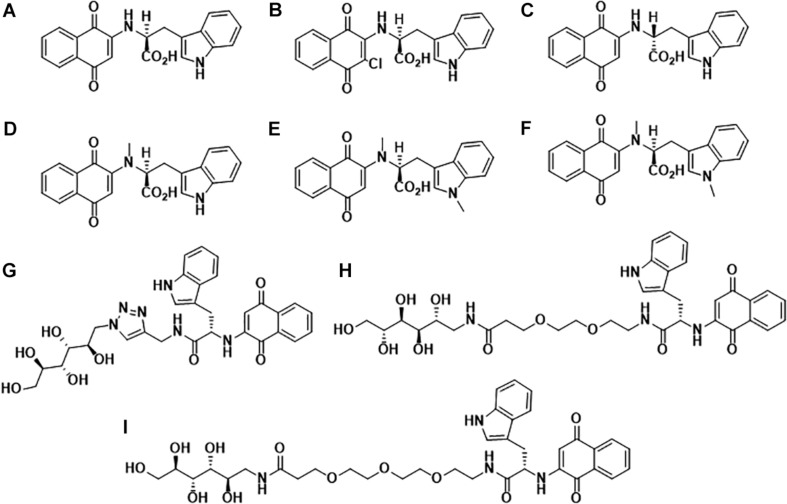
Molecular structures of Naphthoquinone Tryptophan hybrids: **(A)** 1,4-naphthoquinone-2-yl-L-tryptophan (NQTrp), **(B)** Chloro-Naphthoquinone Tryptophan (Cl-NQTrp), **(C)** 1,4-naphthoquinone-2-yl-D-tryptophan (NQ-D-Trp), **(D)** *N*-methylamino-NQTrp (AM), **(E)** *N*-methylindole-NQTrp (IM), **(F)** *N*,*N*-dimethyl-NQTrp (DM), **(G)** Mannitol-Click-NQTrp (MCN), **(H)** Mannitol-2G-NQTrp (M2N), and **(I)** Mannitol-3G-NQTrp (M3N).

In the past decade, our group has extensively worked on the development of Naphthoquinone-based derivatives to modulate amyloid fibrillization. Several Naphthoquinone-Tryptophan (NQTrp) hybrid molecules were developed by conjugating tryptophan (Trp) and Naphthoquinone (NQ) through covalent linkage (Shrestha-Dawadi et al., 1996; Scherzer-Attali et al., 2013). We found that these molecules efficiently inhibited aggregation of various neuronal and non-neuronal amyloidogenic proteins/peptides, as well as disrupted the pre-formed fibrils into non-toxic intermediates. A list of the synthesized NQTrp hybrids is shown in Figure 1. Herein, we briefly review the modulatory effects of NQTrp and its analogs on amyloid fibrillization.

## Naphthoquinone-Tryptophan Hybrids Modulate Aggregation of Neuronal Amyloids

### Amyloid-β Fibrillization

Dementia is an umbrella term associated with the loss of cognitive functions (Savica and Petersen, 2011), and a whopping 50–75% dementia cases are of Alzheimer’s disease (AD) (Qiu et al., 2009), affecting ∼50 million people worldwide^1^. Among the several dysfunctions rationalized for the cause of AD, such as inflammation, oxidative stress, genetics, calcium homeostasis, etc. (Mohandas et al., 2009), one of the widely accepted hypothesis is of the amyloid cascade (Hardy and Selkoe, 2002). The two neuropathological hallmarks of AD are the amyloidogenic deposition of extra-cellular Amyloid β (Aβ peptide) plaques and intra-cellular tangles of the Tau protein (Nisbet et al., 2015). Research in the past two decades has provided a large body of evidence establishing the pathological role of Aβ peptide, where the soluble oligomeric species were found to be the major culprit rather than the mature fibrils (Ahmed et al., 2010; Verma et al., 2015; Sengupta et al., 2016). Therefore, we synthesized and characterized, using *in vitro*, *in silico*, and *in vivo* experiments, NQTrp as an inhibitor for AD-associated Aβ fibrillization.

Upon incubation of Aβ_1–40_ or Aβ_1–42_ with various concentrations of NQTrp, a dose-dependent inhibition of Aβ aggregation was observed using Thioflavin T (ThT) assay. NQTrp significantly inhibited Aβ_1–40_ aggregation even at low molar ratios of 4:1 (Aβ_1–40_: NQTrp), and a similar experiment conducted using Aβ_1–42_ resulted in an IC_50_ value of 50 nM. The results from ThT assay was validated by Transmission Electron Microscopy (TEM) imaging and circular dichroism (CD) spectroscopy, which showed a drastic reduction of the large, broad, ribbon-like fibrils, and a decrease in the β-sheet conformation, respectively with increasing concentration of NQTrp. Interestingly, NQTrp could inhibit Aβ_1–42_ oligomer formation as determined by SDS-PAGE and fluorescence anisotropy assay. The affinity constant (*K*_*d*_) of NQTrp toward early oligomers of Aβ_1–42_ was estimated to be 90 nM. NQTrp has been proposed to stabilize the non-toxic early oligomers and inhibit their further growth into toxic species (Scherzer-Attali et al., 2010).

Furthermore, NQTrp significantly inhibited the cytotoxic effect of the Aβ_1–42_ oligomers and rendered a dose-dependent increase in the viability of Pheochromocytoma cells (PC 12) in culture. The effect of NQTrp on Aβ oligomers and higher-order assemblies was assessed using an animal model. The fruit fly *Drosophila melanogaster* is an established model for various neurodegenerative diseases including AD. Transgenic flies expressing the human Aβ_1–42_ in their central nervous system (CNS) were fed with NQTrp throughout their lifespan. NQTrp treatment prolonged the lifespan of the flies and completely abolished their defective locomotion. Additionally, western blot analysis of the brains of these flies showed a significant reduction in the oligomeric species of Aβ, while immunostaining of the brains of the treated third instar larvae showed a marked decrease in Aβ accumulation (Scherzer-Attali et al., 2010).

**FIGURE 2:**
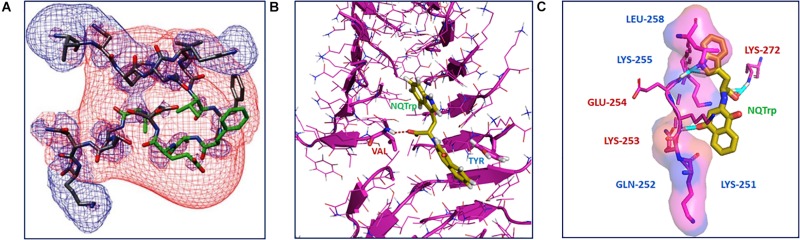
Interaction of NQTrp with amyloidogenic peptides: **(A)** NMR spectroscopy analysis of Aβ peptide with NQTrp. Lowest energy structure generated for Aβ_12__–__28_ with NQTrp (Molar ratio 4:1), where the residues colored in green showed significant deviations upon binding NQTrp. The negative (red) and positive (blue) electrostatic potential distribution for ±2 kT/e are mapped onto the structure (Image reproduced from Scherzer-Attali et al., 2010). **(B)** Complexation of NQTrp with Tau-derived PHF6 peptides in the fibrillar arrangement during disassembly, observed during molecular dynamics simulation (Image reproduced from KrishnaKumar et al., 2018b). **(C)** Putative interaction sites of PAPf39 peptide with NQTrp leading to the inhibiton of its agregation, visualized using molecular docking (Image reproduced from Viswanathan et al., 2018). Interacting residues are shown as sticks.

The mechanism of NQTrp interaction with the Aβ peptides was elucidated using Nuclear Magnetic Resonance (NMR) spectroscopy and Molecular Dynamics (MD) simulations. NMR analysis was performed with NQTrp titrated to Aβ_12__–__28_ and MD simulations were carried out with Aβ_14__–__20_, Aβ_16__–__22_, and Aβ_18__–__24_ in the absence or presence of NQTrp. The outcomes revealed that NQTrp interacted predominantly with the central aromatic core of Aβ by forming hydrogen bonds with the backbone of the Phe20-Glu22 region which contains hydrophobic and charged residues (Figure 2A). This facilitated the NQ ring and indole ring to “clamp” the phenyl ring of either Phe19/Phe20, thus interfering with Aβ association (Scherzer-Attali et al., 2010). A separate simulation study conducted by the Caflisch group with Aβ_12__–__28_ and NQTrp demonstrated that the NQ and indole moieties of NQTrp make appreciable van der Waals interactions with the *N*-terminal stretch of the peptide (residues 13–20). This binding confers electrostatic interactions between the carboxyl group of NQTrp and Aβ residues, i.e., His13, His14, Gln15, and Lys16, which are rich in polar hydrogens (Convertino et al., 2011).

Derreumaux group performed a multi-scale computational study with Aβ_17__–__42_ trimers both in aqueous solution and in the presence of five inhibitors, i.e., 2002-H20, Curcumin, EGCG, NQTrp, and resveratrol. They found NQTrp as the best binder of Aβ. In line with our findings, NQTrp preferentially bound the central hydrophobic core (residues 17–21), and notably with the side chains of Phe19 and Phe20 of Aβ. They proposed that this interaction stabilizes the β-hairpin conformation of one peptide, therefore preventing higher-order β-sheet interactions. However, their MD simulations suggested that NQTrp has multiple binding modes to trimeric Aβ_17__–__42_ conformation (Chebaro et al., 2012). In another study, they performed replica exchange MD simulations of Aβ_1–42_ dimer with two NQTrp molecules and showed that this interaction was very dynamic and multiple, leading to many transient binding sites. In addition to Phe19/Phe20, the hydrophobic residues Leu34/Met35 and hydrophilic/polar residues Arg5, Asp7, Tyr10, His 13, Lys16, and Lys18 were identified as hot spots for NQTrp binding to Aβ_1–42_ (Zhang et al., 2013). Subsequently, they performed simulations of NQTrp with Aβ_1__–__28_ monomer and observed transient interactions with all amino acids, but notably with Asp1, Arg5, Asp7, Tyr10, His12, His13, Lys16, Glu22, Ser26, Arg28, and the hydrophobic patch between Leu17 and Ala21. This finding also showed that NQTrp interactions with Aβ are susceptible to the length of the Aβ peptide. Interestingly, as speculated earlier, NQTrp stabilized the β-hairpin conformation of Aβ hindering its aggregation. In the presence of NQTrp, the population of β-hairpin was reduced by a factor of 1.5, and the population of α-helix in the region 17–24 was increased by a factor of 2 (Tarus et al., 2015). Stabilizing this region and prolonging the α-helical conformation plausibly reduces the conversion to β-sheets, as observed in Aβ amyloid fibrils (Kapurniotu et al., 2003; Tarus et al., 2015). Additionally, side-chain and backbone interactions between NQTrp and Aβ likely limit Aβ–Aβ contacts, thus abrogating fibrillization.

Next, we tested the effect of an analog of NQTrp, named Cl-NQTrp, on *in vitro* aggregation and *in vivo* accumulation of Aβ (Scherzer-Attali et al., 2012a). To that end, monomers of Aβ_1–42_ were incubated with various concentrations of Cl-NQTrp to determine its efficiency to inhibit Aβ oligomerization and specifically the formation of Aβ^∗^56, i.e., a dodecameric form of Aβ with a 56 kDa molecular weight, which was shown to cause memory impairment in AD model mice. As determined by SDS-PAGE and densitometric analysis, Cl-NQTrp reduced the formation of Aβ^∗^56 and concomitantly increased both the intermediate species (∼18 kDa MW, by stabilizing the non-toxic early oligomers) and the monomers. Using NMR, we found that similar to NQTrp (Scherzer-Attali et al., 2010; Chebaro et al., 2012), Cl-NQTrp also binds the aromatic recognition core of Aβ to prevent the formation of oligomers (Scherzer-Attali et al., 2012a). Alongside oligomerization, Cl-NQTrp had an effective dose-dependent inhibitory effect on Aβ_1–40_ fibrillization as determined by ThT assay and TEM imaging. An IC_50_ value of 90 nM was calculated for Cl-NQTrp to inhibit Aβ_1–42_ aggregation. Furthermore, Cl-NQTrp was tested on pre-formed Aβ_1–42_ fibrils to elucidate its disassembly potency. Cl-NQTrp effected a dramatic dose-dependent disassembly of amyloid fibrils as observed by ThT assay and TEM imaging. In transgenic *Drosophila* model expressing Aβ_1–42_ in its CNS, Cl-NQTrp treatment led to a significant amelioration of Aβ-engendered reduced lifespan and defective locomotion. In a 5XFAD AD mouse model, intraperitoneal injection of Cl-NQTrp reduced the level of both the Aβ^∗^56 species (91% reduction) and of total non-soluble Aβ (40% reduction) in their brain, in comparison to vehicle-treated control 5XFAD mice. Importantly, Cl-NQTrp crossed the blood-brain barrier efficiently and did not show toxicity when fed to wild type flies or administered to wild type mice, with no apparent adverse effects on weight gain, mobility or lifespan (Scherzer-Attali et al., 2012a).

Furthermore, we designed and synthesized four NQTrp analogs namely 1,4-naphthoquinone-2-yl-D-tryptophan (NQ-D-Trp), *N*-methylamino-NQTrp (AM), *N*-methylindole-NQTrp (IM), *N*,*N*-dimethyl-NQTrp (DM) and tested their inhibitory effect on Aβ oligomerization and fibrillization (Scherzer-Attali et al., 2013). Similar to NQTrp (Scherzer-Attali et al., 2010), NQ-D-Trp and IM exhibited higher affinity toward Aβ_1–42_ oligomerization with a *K*_*d*_ of 90 nM and reduced Aβ^∗^56 species, but not AM (*K*_*d*_ = 250 nM). On the other hand, NQ-D-Trp had an IC_50_ value of 5–10 nM toward Aβ_1–42_ fibrils, which is lower than the parent NQTrp molecule (IC_50_ = 10–100 nM). However, AM (IC_50_ = 25–50 μM) and IM (IC_50_ = 50 μM) were not effective inhibitors of Aβ_1–42_ fibrillization. Computational analysis showed that the anilinic NH (i.e., the NH linker between the quinone and tryptophan moieties), the quinonic carbonyls, and the carboxylic acid of the NQTrp groups were involved in hydrogen bonds with Aβ. Collectively, we found that NQTrp and NQ-D-Trp had comparable inhibitory activity toward fibrillization and/or oligomerization of Aβ than IM, AM or DM (Scherzer-Attali et al., 2013).

Although NQTrp hybrids facilitated inhibition of Aβ aggregation is well established both *in vitro* and *in vivo*, they are not Aβ-specific inhibitors and thus lack a definite binding site. They may further be improved by side-chain modifications for better binding and providing enhanced inhibitory effects specific to Aβ. Given that there are several modes of action demonstrated for the interaction of Aβ with NQTrp hybrids *in vitro*, the anti-Aβ amyloidogenic activity of NQTrp hybrids *in vivo* is likely to involve other mechanisms in addition to those presented above (Berthoumieu et al., 2015).

### Tau Fibrillization

The Microtubule-Associated Protein Tau (MAPT) is an intra-neuronal protein, which maintains the structural stability of the microtubules (Binder et al., 1985; Kosik, 1993). However, in the AD brain, Tau undergoes abnormal post-translational modifications that render it prone to self-assemble and form β-sheet rich amyloidogenic deposits such as Paired Helical Filaments (PHFs) and Neurofibrillary Tangles (NFTs) that eventually lead to neuronal death (Alonso et al., 2001; Barghorn et al., 2004; Kolarova et al., 2012). Currently, there are no disease-modifying therapeutics for AD or other tauopathies. Therefore, there is an unmet need to screen for and develop compounds to abrogate amyloid Tau aggregation. Aggregation of Tau is believed to be facilitated by two hexapeptide sequences, i.e., ^275^VQIINK^280^ (PHF6^∗^) and ^306^VQIVYK^311^ (PHF6) (Inouye et al., 2006; Ganguly et al., 2015). In line with this, the recently reported cryo-EM structure of Tau filaments suggests that the β-core of the filament comprises of eight β-sheets (β-1 to β-8) spanning the sequence between V^306^ and F^378^, amongst which β-1 is the PHF6 hexapeptide (Fitzpatrick et al., 2017). Hence, we and others have extensively used PHF6 as an *in vitro* proxy model to screen and study inhibitor compounds toward aggregation of full-length (FL) Tau (Zheng et al., 2011; Mohamed et al., 2013; Haj et al., 2018; KrishnaKumar et al., 2018a).

To identify anti-Tau aggregation compounds, we estimated the potency of NQTrp and Cl-NQTrp to inhibit PHF6 aggregation *in vitro* by a variety of biophysical techniques (Frenkel-Pinter et al., 2016, 2017). NQTrp and Cl-NQTrp were found to significantly inhibit PHF6 aggregation in a dose-dependent manner as observed by ThT fluorescence, where the maximum inhibition was attained at 1:5 molar ratio (PHF6: NQTrp/Cl-NQTrp). The outcome of the ThT assay was further validated by CD spectroscopy and TEM imaging, which demonstrated the reduction in the β-sheet content and disappearance of dense, long fibril morphologies of PHF6 upon incubation with NQTrp and Cl-NQTrp, respectively (Frenkel-Pinter et al., 2016, 2017).

The efficacy of NQTrp or Cl-NQTrp was examined *in vivo* using an animal model. Transgenic *Drosophila* overexpressing the human Tau (*h*Tau) protein in the central nervous system or in their retina is widely used for studying tauopathies. The transgenic flies overexpressing *h*Tau were fed with either NQTrp or Cl-NQTrp, mixed in their culture medium, from the beginning of the larval stage onward throughout adult life, and the neurodegenerative phenotypes were scored in comparison with similar flies fed on medium lacking the compounds. We found that treatment with either NQTrp or Cl-NQTrp rescued the eye neurodegenerative phenotype, reduced the accumulation of *h*Tau in the larval eye tissue (25 and 70% reduction for NQTrp and Cl-NQTrp, respectively), reduced Tau hyperphosphorylation, increased the climbing ability, enhanced the lifespan, and led to an overall amelioration of tauopathy-related defects when compared to untreated counterparts (Frenkel-Pinter et al., 2016, 2017).

The AD brain is abundant with mature amyloid aggregates and toxic oligomers of Aβ and Tau (Irvine et al., 2008; Iqbal et al., 2010; Lasagna-Reeves et al., 2011; Brody et al., 2017). Therefore, alongside inhibiting Tau aggregation, it is desirable to reduce the existing load of these amyloid assemblies. To this end, we tested the disassembly efficacy of NQTrp and Cl-NQTrp toward pre-formed PHF6 fibrils and FL-Tau fibrils. Both NQTrp and Cl-NQTrp disassembled pre-formed PHF6 fibrils in a dose-dependent manner as observed by ThS assay (KrishnaKumar et al., 2018b). The maximum disassembly (reduction in ∼40% amyloid content) was attained with 1:5 molar ratio (PHF6: NQTrp/Cl-NQTrp), which was also demonstrated as a marked decrease in the β-sheet content by CD spectroscopy. TEM analysis revealed that treatment of pre-formed PHF6 fibrils by a fivefold molar excess of either NQTrp or Cl-NQTrp resulted in a significant reduction of fibril density and lack of elongated fibrillar structures. Furthermore, the toxicity of PHF6 oligomers, fibrils and disassembled aggregates facilitated by NQTrp or Cl-NQTrp were established with vesicle leakage assay using carboxyfluorescein entrapped Large Unilamellar Vesicles (LUVs) (McLaurin and Chakrabartty, 1996; Williams et al., 2010). We found that the oligomers interacted with the vesicular membrane and ruptured the LUVs causing dye leakage, suggesting that PHF6 oligomers are more toxic than the fibrils. In contrast, pre-formed PHF6 aggregates treated with either NQTrp or Cl-NQTrp caused less dye leakage indicating that the disassembled intermediate products were less toxic than the PHF6 oligomers (KrishnaKumar et al., 2018b).

The interaction of NQTrp and Cl-NQTrp with PHF6 oligomer or fibril was elucidated by MD simulation. We found that NQTrp and Cl-NQTrp interacted with PHF6 in either the oligomer or fibril conformation via hydrogen bonds and π-π stacking. Mechanistically, NQTrp and Cl-NQTrp predominantly interacted with the Val residue of PHF6 by forming hydrogen bonds. Additionally, the aromatic rings of NQ, and Trp of NQTrp, and the aromatic ring of Trp in Cl-NQTrp formed π-π stacking with the side chain of the Tyr residue of PHF6 (Figure 2B; KrishnaKumar et al., 2018b). This is in line with the demonstrated interaction of NQTrp with hydrophobic and polar/charged residues of Aβ determined by NMR and MD simulations, as discussed above (Scherzer-Attali et al., 2010; Convertino et al., 2011; Tarus et al., 2015). Collectively, these interactions caused rupture in the peptide strands by breaking the main-chain hydrogen bonds responsible for the β-sheet formation and disrupted the overall 3D architecture of the oligomer or fibril facilitating disassembly.

We extended our *in vitro* disassembly studies from the PHF6 model system to the FL-Tau protein. Mirroring the results with PHF6, a dose-dependent disassembly of FL-Tau fibrils was effected by both NQTrp and Cl-NQTrp, where the maximum disassembly of fibrils was observed in the presence of 5-fold molar excess. Subsequently, TEM analysis showed that no fibrillary morphologies were detectable in treated samples, unlike the control FL-Tau fibrils, which were not treated with NQTrp or Cl-NQTrp (KrishnaKumar et al., 2018b).

### α-Synuclein Fibrillization

α-Synuclein (α-Syn) is a natively unfolded soluble protein, abundant in the brain, yet its function is not fully understood (Bendor et al., 2013; Emamzadeh, 2016). The aggregation and intra-neuronal inclusions of α-Syn, commonly known as Lewy bodies (LB) or Lewy neurites (LN), are the major cause of various neurological diseases most notably Parkinson’s disease (PD) collectively termed α-synucleinopathies (Fink, 2006). The structure α-Syn is comprised of three domains, an amphipathic domain (N-terminus membrane binding region), a non-amyloidogenic region (NAC) and an acidic domain (C-terminus). The C-terminus domain of α-Syn is highly charged and undergoes phosphorylation at multiple sites, which leads it to misfold and form amyloids. The NAC region of α-Syn is believed to be the most important region in the disease initiation and progression, due to the presence of multiple hydrophobic amino acids, which enhances its aggregation and insolubility (Bendor et al., 2013). However, the mechanism of α-Syn assembly into various fibrillar forms, and how they trigger the demise of neuronal cells remain poorly defined. Recent evidence suggests that the soluble aggregates of α-Syn, referred to as oligomers and protofibrils, are the major cause of neuronal dysfunction (Bendor et al., 2013). Therefore, removing such aggregates would be an important target for disease modification. To this end, we have examined NQTrp hybrids for their ability to modulate α-Syn fibrillization.

Naphthoquinone Tryptophan and several of its analogs have been shown to harbor great potential for inhibition of α-Syn aggregation *in vitro*, and to effectively ameliorate α-Syn induced cytotoxicity. ThT fluorescence assay and TEM imaging results suggested that NQTrp or Cl-NQTrp markedly inhibited *in vitro* aggregation of α-Syn in a dose-dependent manner. However, a higher dose (20 molar excess of α-Syn concentration) of the inhibitor (NQTrp or Cl-NQTrp) was required to achieve significant (∼80%) inhibition (Scherzer-Attali et al., 2012b). Subsequently, to improve the inhibitory efficacy of NQTrp, various conjugate molecules were developed where NQTrp was covalently attached, via click or PEG linker with Mannitol, which by itself was found to moderately inhibit α-Syn aggregation. These Mannitol-NQTrp conjugate molecules (MCN, M2N, and M3N, where the linkage is via either 0, 2, or 3 PEG units, respectively) exhibited excellent inhibitory efficacy toward α-Syn aggregation. The three conjugate molecules substantially inhibited α-Syn aggregation in the presence of lower doses than their parent molecules (Mannitol or NQTrp) or their mixtures, as evident from quantitative ThT assay. A fivefold molar excess of M3N inhibited ∼80% of α-Syn aggregation when compared with NQTrp (∼68%) and Mannitol (∼17%), or their mixture (∼73%) at the same molar ratio, indicating the synergistic effect achieved by their conjugation. This outcome was well corroborated by CD, TEM and Congo-red stained birefringence (Paul et al., 2019). Among these conjugates, M3N was found to be more effective possibly due to the longer linker between NQTrp and Mannitol. We presume that the longer linker confers higher structural flexibility to bind with misfolded assemblies.

M3N was found to be non-cytotoxic to neuronal cells in culture, and it efficiently reduced the cytotoxicity induced by α-Syn aggregates. Importantly, M3N inhibited the formation of α-Syn oligomeric species, which are regarded as more harmful than its higher order aggregates, as evident from the LUV leakage assay. This observation provides an important consideration for drug development (Paul et al., 2019).

## Naphthoquinone-Tryptophan Hybrids Modulate Non-Neuronal Amyloid Aggregation

### Islet Amyloid Polypeptide Fibrillization

Islet amyloid polypeptide (IAPP) is a peptide hormone that is co-secreted with insulin from the pancreatic β-cells islets (Visa et al., 2015). IAPP is prone to self-aggregate and form amyloid deposits which cause the dysfunction of β-cells and pathogenesis of type-2 diabetes mellitus (T2DM). Physiologically, IAPP plays various important roles, e.g., glucose metabolism, glucose homeostasis, glycogen synthesis, control of gastric emptying, and inhibition of glucagon release (Westermark et al., 2011). In healthy individuals, IAPP remains soluble and is stored in granules with insulin, and it is released upon response to the stimuli that lead to insulin secretion. In contrast, insoluble IAPP is known to be responsible for T2DM disease progression (Westermark et al., 2011). The mechanism of IAPP aggregation *in vitro* or *in vivo* to form insoluble amyloid aggregates, which are rich in β-sheet structures remains to be elucidated. However, the smaller assemblies of IAPP, known as oligomers were found to be more toxic to the β-cells than the mature fibrils. Hindering the ability of IAPP to form β-sheet rich aggregates would be a key approach for the inhibition of amyloid formation and disease treatment. Various strategies, including the use of peptides (Paul et al., 2017), nanoparticles (Cabaleiro-Lago et al., 2010), and small molecules (Brahmachari et al., 2017), have been developed for inhibition of IAPP aggregation.

We have demonstrated NQTrp as a potential inhibitor of IAPP aggregation as evident from *in vitro* assays. Quantitative measurement of ThT fluorescence revealed that ∼85% of IAPP amyloid formation was inhibited by only 0.5-fold molar excess. Likewise, 0.5 molar excess of the Cl-NQTrp derivative was able to inhibit ∼75% of IAPP aggregation as evident from ThT assay. NQTrp was found to be slightly more efficacious than Cl-NQTrp (Scherzer-Attali et al., 2012b). The high efficacy of inhibition of IAPP amyloid formation by these molecules is plausibly due to its slow aggregation rate. This may render enough time for the inhibitor molecules to interact or bind with aromatic amino acids in IAPP and inhibit its self-aggregation by steric interference as well as via blocking the intermolecular hydrogen bonding, as mentioned above for inhibition of Aβ and Tau.

The lower order aggregates of IAPP are known to be more toxic than the mature fibrils. Therefore, their detection and characterization hold a key for drawing the mechanism of aggregation, especially at the early stage of self-assembly, and also for the development of potential inhibitors. We utilized a special approach for detecting IAPP dimers, namely the bimolecular fluorescence complementation (BiFC) assay (Bram et al., 2015). This is an artificial genetic system in *Escherichia coli*, in which self-assembled dimers display strong intrinsic fluorescence enabling direct visualization of protein-protein interactions with no need for additional dye. Using BiFC, we could detect the dimeric IAPP and further the inhibition of dimeric IAPP using NQTrp and Cl-NQTrp. We observed that 1:1 molar ratio of NQTrp or Cl-NQTrp inhibited ∼60 and ∼50% of dimeric IAPP, respectively (Bram et al., 2015). The ability of NQTrp hybrids to inhibit the higher order (mature fibrils) aggregation of IAPP as well as to inhibit the dimeric species (oligomers) makes them lead compounds toward IAPP aggregation.

### Prostatic Acid Phosphatase Peptide (PAP_248__–__286_) Fibrillization

PAP_248__–__286_ is a 39 amino acid peptide fragment (henceforth PAPf39) implicated in the acquired immunodeficiency syndrome (AIDS) (Munch et al., 2007; Röcker et al., 2018). PAPf39 forms amyloid fibrils *in vivo*, termed semen-derived enhancer of viral infection (SEVI), which are highly cationic (Roan et al., 2009). These charged amyloids facilitate the attachment of retroviruses, such as human immunodeficiency virus (HIV-1), to host cells by establishing an electrostatic bridge between the negatively charged cell and viral membranes, resulting in the enhancement of viral infection by ∼10^5^ fold (Munch et al., 2007). Therefore, inhibiting the formation of PAPf39 amyloids may be an attractive approach to reduce HIV transmission in AIDS.

We tested the ability of NQTrp to inhibit PAPf39 aggregation using ThT assay and found that it significantly inhibited aggregation of this amyloid *in vitro* in a dose-dependent manner. Maximum inhibitory activity of NQTrp was observed at 1:1 molar ratio (PAPf39: NQTrp). The ThT assay results were further validated using complementary spectroscopic methods including ANS binding and Congo red birefringence assay. At an equimolar ratio (PAPf39: NQTrp), ANS emission spectra post-aggregation overlapped with that of monomeric PAPf39, suggesting that NQTrp stabilized the native conformation of the peptide monomer. Furthermore, the control PAPf39, i.e., in the absence of NQTrp, developed a characteristic apple-green birefringence under cross-polarized light upon incubation with Congo red, which completely disappeared at 1:1 molar ratio (PAPf39: NQTrp). Morphology of the PAPf39 fibrils in the absence or presence of NQTrp was visualized using TEM imaging. The untreated PAPf39 fibrils appeared mature, long, and dense. However, at 1:1 molar ratio (PAPf39: NQTrp), the density of the fibrils was substantially reduced, and no elongated fibril morphologies were visible. Notably, inhibition of PAPf39 aggregation by NQTrp resulted in non-toxic lower MW intermediates as demonstrated by the LUV leakage assay (Viswanathan et al., 2018).

Binding of NQTrp with PAPf39 monomers was evaluated using isothermal titration calorimetry (ITC) measurements. We found the binding to be spontaneous and the interaction between NQTrp and PAPf39 was an enthalpy-driven process, i.e., the interaction was preferentially due to hydrogen bonding and electrostatic interactions (Viswanathan et al., 2018). Molecular docking was performed to identify the putative amino acid residues interacting with NQTrp. NQTrp was found to interact with two regions in PAPf39 that are enriched with charged and polar residues, i.e., Region 1 – Lys251 to Leu258, and Region 2 – Met271 to Arg273, which was in line with EGCG-PAPf39 binding sites as determined using NMR (Popovych et al., 2012). NQTrp formed hydrogen bonds with Lys253, Glu254, and Lys272 and facilitated hydrophobic contacts with Lys251, Gln252, Lys255, and Leu258 (Figure 2C), a binding mechanism similar to NQTrp-Aβ and NQTrp-PHF6 interaction (Scherzer-Attali et al., 2010; Convertino et al., 2011; KrishnaKumar et al., 2018b).

### Calcitonin, Insulin, and Lysozyme Fibrillization

We evaluated the inhibitory effect of NQTrp and Cl-NQTrp toward aggregation of Calcitonin, Insulin and Lysozyme aggregation (Scherzer-Attali et al., 2012b), which form amyloids *in vivo* and are implicated in medullar carcinoma of the thyroid, insulin injection amyloidosis and hereditary systemic amyloidosis, respectively (Pepys et al., 1993; Westermark et al., 2005; Guo et al., 2015). NQTrp was found to be more efficient than Cl-NQTrp in inhibiting aggregation of Calcitonin as determined by ThT assay. At lower molar ratio of 2:1 (Calcitonin: NQTrp/Cl-NQTrp), NQTrp inhibited 94%, whereas Cl-NQTrp inhibited 30% amyloid formation. However, at a higher molar ratio of 1:20, both compounds completely inhibited Calcitonin fibrillization (Scherzer-Attali et al., 2012b). Likewise, incubation of NQTrp or Cl-NQTrp with Insulin resulted in substantial amyloid inhibition (70–80%) at 2:1 molar ratio (Insulin: NQTrp/Cl-NQTrp), and up to 95–98% inhibition at a higher molar ratio of 1:20 (Scherzer-Attali et al., 2012b). In contrast, NQTrp and Cl-NQTrp were less effective as inhibitors of Lysozyme aggregation. Incubation of NQTrp or Cl-NQTrp with Lysozyme inhibited only 10–20% amyloids at 2:1 molar ratio (Lysozyme: NQTrp/Cl-NQTrp), and 50% inhibition at higher molar ratio of 1:20 for both compounds. The outcomes of ThT assay was well corroborated by TEM imaging of fibrils (Scherzer-Attali et al., 2012b).

## Conclusion and Further Scope

**FIGURE 3:**
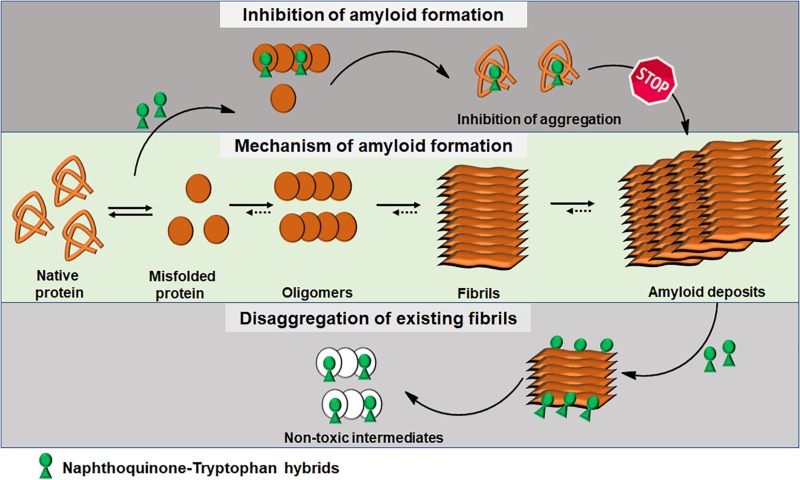
Schematics of amyloid aggregation and its inhibition/disaggregation by Naphthoquinone-Tryptophan hybrid molecules.

Integrating experimental and computational analyses, we have demonstrated the modulatory effect of NQTrp hybrids toward the aggregation of neuronal and non-neuronal amyloids. NQTrp and its analogs were effective inhibitors of amyloid aggregation as well as disrupted the pre-formed amyloid assemblies (Figure 3). The hybrid molecules are non-toxic to cells in culture and do not have apparent adverse effects on animal models used for neurodegeneration studies. In those models, the compounds were administered from early stages of the lifecycle, presumably before significant accumulation of amyloids has taken place. It remains to be examined what will be the effect of treating the animal models at a later stage in life after pathology has started to develop, which would better simulate the situation when treating the patient. Thus, the NQTrp scaffold emerges as a promising small molecule backbone, which can further be modified for targeted inhibition of specific amyloids. We are currently developing NQTrp analogs by conjugation with neurotransmitters, sugars, varying the length of the linker between them, and testing these compounds on Aβ, Tau, α-synuclein, IAPP and γ-D-crystallin aggregation. It is worth bearing in mind that these complex diseases may have multiple causes hence, effective therapy may require multi-targeting.

## Author Contributions

GV and DS structured the manuscript. GV compiled Aβ, Tau, PAP_248__–__286_, Calcitonin, Insulin, and Lysozyme sections. AP compiled α-Syn and IAPP sections. EG and DS overviewed the text. All authors read and approved the manuscript.

## Conflict of Interest

The authors declare that the research was conducted in the absence of any commercial or financial relationships that could be construed as a potential conflict of interest.

## Footnotes

1 https://www.alz.co.uk/research/statistics

## References

[B1] Ahmed M. Davis J. Aucoin D. Sato T. Ahuja S. Aimoto S. (2010). Structural conversion of neurotoxic amyloid-beta(1-42) oligomers to fibrils. *Nat. Struct. Mol. Biol.* 17 561–567. 10.1038/nsmb.1799 20383142 PMC2922021

[B2] Alonso A. Zaidi T. Novak M. Grundke-Iqbal I. Iqbal K. (2001). Hyperphosphorylation induces self-assembly of tau into tangles of paired helical filaments/straight filaments. *Proc. Natl. Acad. Sci. U.S.A.* 98 6923–6928. 10.1073/pnas.121119298 11381127 PMC34454

[B3] Barghorn S. Davies P. Mandelkow E. (2004). Tau paired helical filaments from Alzheimer’s disease brain and assembled in vitro are based on beta-structure in the core domain. *Biochemistry* 43 1694–1703. 10.1021/bi0357006 14769047

[B4] Bendor J. T. Logan T. P. Edwards R. H. (2013). The function of alpha-synuclein. *Neuron* 79 1044–1066. 10.1016/j.neuron.2013.09.004 24050397 PMC3866954

[B5] Bermejo-Bescos P. Martin-Aragon S. Jimenez-Aliaga K. L. Ortega A. Molina M. T. Buxaderas E. (2010). In vitro antiamyloidogenic properties of 1,4-naphthoquinones. *Biochem. Biophys. Res. Commun.* 400 169–174. 10.1016/j.bbrc.2010.08.038 20709023

[B6] Berthoumieu O. Nguyen P. H. Castillo-Frias M. P. Ferre S. Tarus B. Nasica-Labouze J. (2015). Combined experimental and simulation studies suggest a revised mode of action of the anti-Alzheimer disease drug NQ-Trp. *Chemistry* 21 12657–12666. 10.1002/chem.201500888 26179053

[B7] Binder L. I. Frankfurter A. Rebhun L. I. (1985). The distribution of tau in the mammalian central nervous system. *J. Cell Biol.* 101 1371–1378. 10.1083/jcb.101.4.1371 3930508 PMC2113928

[B8] Bisaglia M. Tosatto L. Munari F. Tessari I. de Laureto P. P. Mammi S. (2010). Dopamine quinones interact with alpha-synuclein to form unstructured adducts. *Biochem. Biophys. Res. Commun.* 394 424–428. 10.1016/j.bbrc.2010.03.044 20226175

[B9] Brahmachari S. Paul A. Segal D. Gazit E. (2017). Inhibition of amyloid oligomerization into different supramolecular architectures by small molecules: mechanistic insights and design rules. *Future Med. Chem.* 9 797–810. 10.4155/fmc-2017-0026 28485623

[B10] Bram Y. Lampel A. Shaltiel-Karyo R. Ezer A. Scherzer-Attali R. Segal D. (2015). Monitoring and targeting the initial dimerization stage of amyloid self-assembly. *Angew. Chem. Int. Ed. Engl.* 54 2062–2067. 10.1002/anie.201408744 25533189

[B11] Brody D. L. Jiang H. Wildburger N. Esparza T. J. (2017). Non-canonical soluble amyloid-beta aggregates and plaque buffering: controversies and future directions for target discovery in Alzheimer’s disease. *Alzheimers Res. Ther.* 9:62. 10.1186/s13195-017-0293-3 28818091 PMC5561579

[B12] Cabaleiro-Lago C. Lynch I. Dawson K. A. Linse S. (2010). Inhibition of IAPP and IAPP(20-29) fibrillation by polymeric nanoparticles. *Langmuir* 26 3453–3461. 10.1021/la902980d 20017535

[B13] Chebaro Y. Jiang P. Zang T. Mu Y. Nguyen P. H. Mousseau N. (2012). Structures of Abeta17-42 trimers in isolation and with five small-molecule drugs using a hierarchical computational procedure. *J. Phys. Chem. B* 116 8412–8422. 10.1021/jp2118778 22283547

[B14] Convertino M. Pellarin R. Catto M. Carotti A. Caflisch A. (2009). 9,10-Anthraquinone hinders beta-aggregation: how does a small molecule interfere with Abeta-peptide amyloid fibrillation? *Protein Sci.* 18 792–800. 10.1002/pro.87 19309732 PMC2762591

[B15] Convertino M. Vitalis A. Caflisch A. (2011). Disordered binding of small molecules to Abeta(12-28). *J. Biol. Chem.* 286 41578–41588. 10.1074/jbc.M111.285957 21969380 PMC3308868

[B16] Emamzadeh F. N. (2016). Alpha-synuclein structure, functions, and interactions. *J. Res. Med. Sci.* 21:29. 10.4103/1735-1995.181989 27904575 PMC5122110

[B17] Fink A. L. (2006). The aggregation and fibrillation of alpha-synuclein. *Acc. Chem. Res.* 39 628–634. 10.1021/ar050073t 16981679

[B18] Fitzpatrick A. W. P. Falcon B. He S. Murzin A. G. Murshudov G. Garringer H. J. (2017). Cryo-EM structures of tau filaments from Alzheimer’s disease. *Nature* 547 185–190. 10.1038/nature23002 28678775 PMC5552202

[B19] Frenkel-Pinter M. Tal S. Scherzer-Attali R. Abu-Hussien M. Alyagor I. Eisenbaum T. (2016). Naphthoquinone-tryptophan hybrid inhibits aggregation of the tau-derived peptide PHF6 and reduces neurotoxicity. *J. Alzheimers Dis.* 51 165–178. 10.3233/JAD-150927 26836184

[B20] Frenkel-Pinter M. Tal S. Scherzer-Attali R. Abu-Hussien M. Alyagor I. Eisenbaum T. (2017). Cl-NQTrp alleviates tauopathy symptoms in a model organism through the inhibition of tau aggregation-engendered toxicity. *Neurodegener. Dis.* 17 73–82. 10.1159/000448518 27760426

[B21] Ganguly P. Do T. D. Larini L. LaPointe N. E. Sercel A. J. Shade M. F. (2015). Tau assembly: the dominant role of PHF6 (VQIVYK) in microtubule binding region repeat R3. *J. Phys. Chem. B* 119 4582–4593. 10.1021/acs.jpcb.5b00175 25775228 PMC4428543

[B22] Gong H. He Z. Peng A. Zhang X. Cheng B. Sun Y. (2014). Effects of several quinones on insulin aggregation. *Sci. Rep.* 4:5648. 10.1038/srep05648 25008537 PMC4090620

[B23] Guo C. Ma L. Zhao Y. Peng A. Cheng B. Zhou Q. (2015). Inhibitory effects of magnolol and honokiol on human calcitonin aggregation. *Sci. Rep.* 5:13556. 10.1038/srep13556 26324190 PMC4555095

[B24] Haj E. Losev Y. Guru KrishnaKumar V. Pichinuk E. Engel H. Raveh A. (2018). Integrating in vitro and in silico approaches to evaluate the “dual functionality” of palmatine chloride in inhibiting and disassembling Tau-derived VQIVYK peptide fibrils. *Biochim. Biophys. Acta* 1862 1565–1575. 10.1016/j.bbagen.2018.04.001 29634991

[B25] Hardy J. Selkoe D. J. (2002). The amyloid hypothesis of Alzheimer’s disease: progress and problems on the road to therapeutics. *Science* 297 353–356. 10.1126/science.1072994 12130773

[B26] Inouye H. Sharma D. Goux W. J. Kirschner D. A. (2006). Structure of core domain of fibril-forming PHF/Tau fragments. *Biophys. J.* 90 1774–1789. 10.1529/biophysj.105.070136 16339876 PMC1367326

[B27] Iqbal K. Liu F. Gong C.-X. Grundke-Iqbal I. (2010). Tau in Alzheimer disease and related tauopathies. *Curr. Alzheimer Res.* 7 656–664. 10.2174/156720510793611592 20678074 PMC3090074

[B28] Irvine G. B. El-Agnaf O. M. Shankar G. M. Walsh D. M. (2008). Protein aggregation in the brain: the molecular basis for Alzheimer’s and Parkinson’s diseases. *Mol. Med.* 14 451–464. 10.2119/2007-00100.Irvine 18368143 PMC2274891

[B29] Kapurniotu A. Buck A. Weber M. Schmauder A. Hirsch T. Bernhagen J. (2003). Conformational restriction via cyclization in beta-amyloid peptide Abeta(1-28) leads to an inhibitor of Abeta(1-28) amyloidogenesis and cytotoxicity. *Chem. Biol.* 10 149–159. 10.1016/s1074-5521(03)00022-x 12618187

[B30] Kim J. Kobayashi M. Fukuda M. Ogasawara D. Kobayashi N. Han S. (2010). Pyrroloquinoline quinone inhibits the fibrillation of amyloid proteins. *Prion* 4 26–31. 10.4161/pri.4.1.10889 20083898 PMC2850417

[B31] Kobayashi M. Kim J. Kobayashi N. Han S. Nakamura C. Ikebukuro K. (2006). Pyrroloquinoline quinone (PQQ) prevents fibril formation of alpha-synuclein. *Biochem. Biophys. Res. Commun.* 349 1139–1144. 10.1016/j.bbrc.2006.08.144 16962995

[B32] Kolarova M. Garcia-Sierra F. Bartos A. Ricny J. Ripova D. (2012). Structure and pathology of tau protein in Alzheimer disease. *Int. J. Alzheimers Dis.* 2012 731526. 10.1155/2012/731526 22690349 PMC3368361

[B33] Kosik K. S. (1993). The molecular and cellular biology of tau. *Brain Pathol.* 3 39–43. 10.1111/j.1750-3639.1993.tb00724.x 8269082

[B34] KrishnaKumar V. G. Baweja L. Ralhan K. Gupta S. (2018a). Carbamylation promotes amyloidogenesis and induces structural changes in Tau-core hexapeptide fibrils. *Biochim. Biophys. Acta. Gen. Subj.* 1862 2590–2604. 10.1016/j.bbagen.2018.07.030 30071272

[B35] KrishnaKumar V. G. Paul A. Gazit E. Segal D. (2018b). Mechanistic insights into remodeled Tau-derived PHF6 peptide fibrils by naphthoquinone-tryptophan hybrids. *Sci. Rep.* 8:71. 10.1038/s41598-017-18443-2 29311706 PMC5758761

[B36] Lasagna-Reeves C. A. Castillo-Carranza D. L. Sengupta U. Clos A. L. Jackson G. R. Kayed R. (2011). Tau oligomers impair memory and induce synaptic and mitochondrial dysfunction in wild-type mice. *Mol. Neurodegener.* 6:39. 10.1186/1750-1326-6-39 21645391 PMC3224595

[B37] Li G. Sun W. Li J. Jia F. Hong L. Wang R. (2015). Organocatalytic enantioselective formal arylation of azlactones using quinones as the aromatic partner. *Chem. Commun.* 51 11280–11282. 10.1039/c5cc03677a 26083993

[B38] Li J. Zhu M. Rajamani S. Uversky V. N. Fink A. L. (2004). Rifampicin inhibits alpha-synuclein fibrillation and disaggregates fibrils. *Chem. Biol.* 11 1513–1521. 10.1016/j.chembiol.2004.08.025 15556002

[B39] Lieu V. H. Wu J. W. Wang S. S.-S. Wu C.-H. (2007). Inhibition of amyloid fibrillization of hen egg-white lysozymes by rifampicin and p-benzoquinone. *Biotechnol. Prog.* 23 698–706. 10.1021/bp060353n 17492832

[B40] McLaurin J. Chakrabartty A. (1996). Membrane disruption by Alzheimer beta-amyloid peptides mediated through specific binding to either phospholipids or gangliosides. Implications for neurotoxicity. *J. Biol. Chem.* 271 26482–26489. 10.1074/jbc.271.43.26482 8900116

[B41] Mohamed T. Hoang T. Jelokhani-Niaraki M. Rao P. P. N. (2013). Tau-derived-hexapeptide (306)VQIVYK(311) aggregation inhibitors: nitrocatechol moiety as A pharmacophore in drug design. *ACS Chem. Neurosci.* 4 1559–1570. 10.1021/cn400151a 24007550 PMC3867965

[B42] Mohandas E. Rajmohan V. Raghunath B. (2009). Neurobiology of Alzheimer’s disease. *Indian J. Psychiatry* 51 55–61. 10.4103/0019-5545.44908 19742193 PMC2738403

[B43] Munch J. Rucker E. Standker L. Adermann K. Goffinet C. Schindler M. (2007). Semen-derived amyloid fibrils drastically enhance HIV infection. *Cell* 131 1059–1071. 10.1016/j.cell.2007.10.014 18083097

[B44] Nisbet R. M. Polanco J.-C. Ittner L. M. Götz J. (2015). Tau aggregation and its interplay with amyloid-β. *Acta Neuropathol.* 129 207–220. 10.1007/s00401-014-1371-2 25492702 PMC4305093

[B45] Nohl H. Jordan W. Youngman R. J. (1986). Quinones in biology: functions in electron transfer and oxygen activation. *Adv. Free Radic. Biol. Med.* 2 211–279. 10.1016/S8755-9668(86)80030-8

[B46] Paul A. Kalita S. Kalita S. Sukumar P. Mandal B. (2017). Disaggregation of amylin aggregate by novel conformationally restricted aminobenzoic acid containing α/β and α/γ hybrid peptidomimetics. *Sci. Rep.* 7:40095. 10.1038/srep40095 28054630 PMC5214534

[B47] Paul A. Zhang B.-D. Mohapatra S. Li G. Li Y.-M. Gazit E. (2019). Novel mannitol-based small molecules for inhibiting aggregation of alpha-synuclein amyloids in Parkinson’s disease. *Front. Mol. Biosci.* 6:16 10.3389/fmolb.2019.00016 PMC6438916 30968030

[B48] Pepys M. B. Hawkins P. N. Booth D. R. Vigushin D. M. Tennent G. A. Soutar A. K. (1993). Human lysozyme gene mutations cause hereditary systemic amyloidosis. *Nature* 362 553–557. 10.1038/362553a0 8464497

[B49] Pickhardt M. Gazova Z. von Bergen M. Khlistunova I. Wang Y. Hascher A. (2005). Anthraquinones inhibit tau aggregation and dissolve Alzheimer’s paired helical filaments in vitro and in cells. *J. Biol. Chem.* 280 3628–3635. 10.1074/jbc.M410984200 15525637

[B50] Pinto A. V. de Castro S. L. (2009). The trypanocidal activity of naphthoquinones: a review. *Molecules* 14 4570–4590. 10.3390/molecules14114570 19924086 PMC6255437

[B51] Popovych N. Brender J. R. Soong R. Vivekanandan S. Hartman K. Basrur V. (2012). Site specific interaction of the polyphenol EGCG with the SEVI amyloid precursor peptide PAP(248-286). *J. Phys. Chem. B* 116 3650–3658. 10.1021/jp2121577 22360607 PMC3310975

[B52] Qiu C. Kivipelto M. von Strauss E. (2009). Epidemiology of Alzheimer’s disease: occurrence, determinants, and strategies toward intervention. *Dialogues Clin. Neurosci.* 11 111–128. 19585947 10.31887/DCNS.2009.11.2/cqiu PMC3181909

[B53] Roan N. R. Münch J. Arhel N. Mothes W. Neidleman J. Kobayashi A. (2009). The cationic properties of SEVI underlie its ability to enhance human immunodeficiency virus infection. *J. Virol.* 83 73–80. 10.1128/JVI.01366-08 18945786 PMC2612336

[B54] Röcker A. Roan N. R. Yadav J. K. Fändrich M. Münch J. (2018). Structure, function and antagonism of semen amyloids. *Chem. Commun.* 54 7557–7569. 10.1039/C8CC01491D 29873340 PMC6033663

[B55] Rohr J. Thiericke R. (1992). Angucycline group antibiotics. *Nat. Prod. Rep.* 9 103–137. 1620493 10.1039/np9920900103

[B56] Savica R. Petersen R. C. (2011). Prevention of dementia. *Psychiatr. Clin. North Am.* 34 127–145. 10.1016/j.psc.2010.11.006 21333844 PMC4634887

[B57] Scherzer-Attali R. Convertino M. Pellarin R. Gazit E. Segal D. Caflisch A. (2013). Methylations of tryptophan-modified naphthoquinone affect its inhibitory potential toward Abeta aggregation. *J. Phys. Chem. B* 117 1780–1789. 10.1021/jp309066p 23259849

[B58] Scherzer-Attali R. Farfara D. Cooper I. Levin A. Ben-Romano T. Trudler D. (2012a). Naphthoquinone-tyrptophan reduces neurotoxic Abeta^∗^56 levels and improves cognition in Alzheimer’s disease animal model. *Neurobiol. Dis.* 46 663–672. 10.1016/j.nbd.2012.03.005 22449754

[B59] Scherzer-Attali R. Shaltiel-Karyo R. Adalist Y. H. Segal D. Gazit E. (2012b). Generic inhibition of amyloidogenic proteins by two naphthoquinone-tryptophan hybrid molecules. *Proteins* 80 1962–1973. 10.1002/prot.24080 22488522

[B60] Scherzer-Attali R. Pellarin R. Convertino M. Frydman-Marom A. Egoz-Matia N. Peled S. (2010). Complete phenotypic recovery of an Alzheimer’s disease model by a quinone-tryptophan hybrid aggregation inhibitor. *PLoS One* 5:e11101. 10.1371/journal.pone.0011101 20559435 PMC2885425

[B61] Sengupta U. Nilson A. N. Kayed R. (2016). The role of Amyloid-β oligomers in toxicity, propagation, and immunotherapy. *EBioMedicine* 6 42–49. 10.1016/j.ebiom.2016.03.035 27211547 PMC4856795

[B62] Shrestha-Dawadi P. B. Bittner S. Fridkin M. Rahimpour S. (1996). On the synthesis of Naphthoquinonyl hetrocyclic amino acids. *Synthesis* 12 1468–1472. 10.1055/s-1996-4417

[B63] Sunassee S. N. Davies-Coleman M. T. (2012). Cytotoxic and antioxidant marine prenylated quinones and hydroquinones. *Nat. Prod. Rep.* 29 513–535. 10.1039/c2np00086e 22382850

[B64] Tarus B. Nguyen P. H. Berthoumieu O. Faller P. Doig A. J. Derreumaux P. (2015). Molecular structure of the NQTrp inhibitor with the Alzheimer Abeta1-28 monomer. *Eur. J. Med. Chem.* 91 43–50. 10.1016/j.ejmech.2014.07.002 25011560

[B65] Tomiyama T. Shoji A. Kataoka K. Suwa Y. Asano S. Kaneko H. (1996). Inhibition of amyloid beta protein aggregation and neurotoxicity by rifampicin. Its possible function as a hydroxyl radical scavenger. *J. Biol. Chem.* 271 6839–6844. 10.1074/jbc.271.12.6839 8636108

[B66] Verma M. Vats A. Taneja V. (2015). Toxic species in amyloid disorders: oligomers or mature fibrils. *Ann. Indian Acad. Neurol.* 18 138–145. 10.4103/0972-2327.144284 26019408 PMC4445186

[B67] Visa M. Alcarraz-Vizan G. Montane J. Cadavez L. Castano C. Villanueva-Penacarrillo M. L. (2015). Islet amyloid polypeptide exerts a novel autocrine action in beta-cell signaling and proliferation. *FASEB J.* 29 2970–2979. 10.1096/fj.15-270553 25808537

[B68] Viswanathan G. K. Mohapatra S. Paul A. Arad E. Jelinek R. Gazit E. (2018). Inhibitory effect of naphthoquinone-tryptophan hybrid towards aggregation of PAP f39 semen amyloid. *Molecules* 23:E3279. 10.3390/molecules23123279 30544943 PMC6320874

[B69] Westermark P. Andersson A. Westermark G. T. (2011). Islet amyloid polypeptide, islet amyloid, and diabetes mellitus. *Physiol. Rev.* 91 795–826. 10.1152/physrev.00042.2009 21742788

[B70] Westermark P. Benson M. D. Buxbaum J. N. Cohen A. S. Frangione B. Ikeda S.-I. (2005). Amyloid: toward terminology clarification. Report from the nomenclature committee of the international society of amyloidosis. *Amyloid Int. J. Exp. Clin. Investig.* 12 1–4. 10.1080/13506120500032196 16076605

[B71] Williams T. L. Day I. J. Serpell L. C. (2010). The effect of Alzheimer’s Abeta aggregation state on the permeation of biomimetic lipid vesicles. *Langmuir* 26 17260–17268. 10.1021/la101581g 20923185

[B72] Zhang J.-J. Zhang R.-F. Meng X.-K. (2009). Protective effect of pyrroloquinoline quinone against Abeta-induced neurotoxicity in human neuroblastoma SH-SY5Y cells. *Neurosci. Lett.* 464 165–169. 10.1016/j.neulet.2009.08.037 19699263

[B73] Zhang T. Xu W. Mu Y. Derreumaux P. (2013). Atomic and dynamic insights into the beneficial effect of the 1,4-naphthoquinon-2-yl-L-tryptophan inhibitor on Alzheimer’s Aβ1-42 dimer in terms of aggregation and toxicity. *ACS Chem. Neurosci.* 5 148–159. 10.1021/cn400197x 24246047 PMC3930991

[B74] Zheng J. Liu C. Sawaya M. R. Vadla B. Khan S. Woods R. J. (2011). Macrocyclic beta-sheet peptides that inhibit the aggregation of a tau-protein-derived hexapeptide. *J. Am. Chem. Soc.* 133 3144–3157. 10.1021/ja110545h 21319744 PMC3048834

